# CEACAM1 expression by immunohistochemistry in B-cell lymphomas and plasma cell myeloma

**DOI:** 10.1093/ajcp/aqag078

**Published:** 2026-06-29

**Authors:** V Rakesh Sethapati, Vu Ngo, Alexey Danilov, Tycel Phillips, Geoffrey Shouse, Alex Herrera, Anamarija M Perry, Aimin Li, Larry Kwak, Dennis D Weisenburger, Wing C Chan, Joo Y Song

**Affiliations:** Department of Pathology, University of Arkansas for Medical Sciences, Little Rock, AR, United States; Department of Pathology, City of Hope Medical Center, Duarte, CA, United States; Department of Hematology and Hematopoietic Cell Transplantation, City of Hope Medical Center, Duarte, CA, United States; Department of Hematology and Hematopoietic Cell Transplantation, City of Hope Medical Center, Duarte, CA, United States; Department of Hematology and Hematopoietic Cell Transplantation, City of Hope Medical Center, Duarte, CA, United States; Department of Hematology and Hematopoietic Cell Transplantation, City of Hope Medical Center, Duarte, CA, United States; Department of Pathology, University of Michigan, Ann Arbor, MI, United States; Department of Pathology, City of Hope Medical Center, Duarte, CA, United States; Department of Hematology and Hematopoietic Cell Transplantation, City of Hope Medical Center, Duarte, CA, United States; Department of Pathology, Microbiology, and Immunology, University of Nebraska Medical Center, Omaha, NE, United States; Department of Pathology, City of Hope Medical Center, Duarte, CA, United States; Department of Pathology, City of Hope Medical Center, Duarte, CA, United States

**Keywords:** CEACAM1, lymphoma, multiple myeloma, therapeutic targets

## Abstract

**Objectives:**

Mantle cell lymphoma (MCL) is an aggressive B-cell lymphoma with limited curative treatment options. Carcinoembryonic antigen cell adhesion molecule 1 (CEACAM1) has recently been implicated in B-cell receptor signaling in MCL. We evaluated CEACAM1 expression by immunohistochemistry across MCL and other mature B-cell neoplasms, including small lymphocytic lymphoma (SLL), marginal zone lymphoma (MZL), diffuse large B-cell lymphoma (DLBCL), and multiple myeloma (MM).

**Methods:**

We retrospectively analyzed 164 lymphoma cases, 35 MM cases, and 2 plasmacytomas. CEACAM1 expression was assessed on tissue microarrays by immunohistochemistry and scored using a prespecified dichotomous cutoff. In DLBCL, CEACAM1 status was correlated with cell-of-origin classification and targeted mutational data.

**Results:**

CEACAM1 was expressed in 27 of 30 MCL cases (90%), higher than in the other lymphoma subtypes combined (35%; *P* < .0001). Expression was also seen in SLL (20/30, 67%), MM (23/35, 66%), MZL (8/22, 36%), and DLBCL (19/82, 23%). In DLBCL, expression was more frequent in germinal center B-cell subtype than activated B-cell subtype cases (30% vs 13%; *P* = .023). Within sequenced DLBCL cases, CEACAM1 expression was associated with *BCL2* and *TNFRSF14* mutations. In MM, CEACAM1 expression was not significantly associated with recurrent cytogenetic abnormalities, although a nonsignificant trend toward higher expression in t(11;14) cases (75% vs 53%, Fisher exact *P* = .28).

**Conclusions:**

CEACAM1 is frequently expressed in MCL and in subsets of other mature B-cell neoplasms and MM. These findings support further investigation of CEACAM1 as a biologically relevant marker and potential therapeutic target in selected lymphoid malignancies.

KEY POINTS

- CEACAM1 expression is most frequent in mantle cell lymphoma and is also present in subsets of small lymphocytic lymphoma, marginal zone lymphoma, diffuse large B-cell lymphoma, and multiple myeloma.
- In diffuse large B-cell lymphoma, CEACAM1 expression is more common in germinal center B-cell subtype cases and is associated with *BCL2* and *TNFRSF14* mutations.
- CEACAM1 immunohistochemistry may help define the distribution of this biologically relevant marker across mature B-cell neoplasms.

## INTRODUCTION

Mantle cell lymphoma (MCL) is a mature B-cell lymphoma that accounts for approximately 2.5% to 6% of non-Hodgkin lymphomas. It remains clinically aggressive, with most patients eventually relapsing despite contemporary chemoimmunotherapy and targeted approaches.^1‐6^

Xavier et al^3^ recently identified carcinoembryonic antigen cell adhesion molecule 1 (CEACAM1) as a central driver of oncogenic BCR signaling in MCL. Using a combination of genome-wide CRISPR screens, gene expression profiling, and BCR signal transduction studies, that study demonstrated that CEACAM1 facilitates BCR signaling by localizing membrane microdomains (lipid rafts) and recruiting SYK kinase to the BCR complex. CEACAM1 is a primordial member of the carcinoembryonic antigen (CEA) family of immunoglobulin-like transmembrane proteins and is expressed in B and T lymphocytes, natural killer cells, granulocytes, epithelial cells, and certain endothelial cells.^4^ CEACAM1 acts as a cell membrane receptor to transduce extracellular signals into the cytoplasmic component. Depending on the presence of a full-length (long) or an alternatively spliced (short) cytoplasmic tail, CEACAM1 will impart inhibitory or noninhibitory (activating) signals, respectively.^3^^,^^5^ Mechanistic studies revealed that CEACAM1 plays a dual role in BCR signaling, acting as an activator in cells with high CEACAM1 expression but functioning as an inhibitor in cells with low CEACAM1 expression by preferentially binding to SHIP-1 phosphatase. CEACAM1 appears to affect a broad range of functions, including apoptosis, angiogenesis, cell proliferation, cell motility, and immune T-cell tolerance.^5^^,^^7^^,^^8^

In the present study, CEACAM1 membranous expression was evaluated across mature B-cell lymphomas and plasma cell myeloma by immunohistochemistry using standardized scoring criteria. Associations with diffuse large B-cell lymphoma (DLBCL) cell-of-origin classification and recurrent somatic mutations were also assessed. Because CEACAM1 expression in DLBCL was previously reported by Xavier et al,^3^ the DLBCL cohort is retained in the present study to allow correlation of CEACAM1 expression with cell-of-origin, recurrent somatic mutations and overall survival in the sequenced subset. The classic Hodgkin lymphoma and follicular lymphoma cohorts from that prior study are not re-presented here. The present study additionally evaluates small lymphocytic lymphoma (SLL), marginal zone lymphoma (MZL), and an expanded multiple myeloma (MM) cohort with cytogenetic correlations.

## MATERIALS AND METHODS

### Patients and cases

We retrospectively selected MCL (n = 30), DLBCL (n = 82), MZL (n = 22; 12 nodal MZLs, 8 extranodal MZLs, 1 transformed MZL, and 1 cutaneous MZL), SLL (n = 30), MM (n = 35), and plasmacytoma (n = 2) cases from City of Hope National Medical Center between 2000 and 2015. Cases were selected and rereviewed for accuracy of diagnosis, according to the International Consensus Classification^9^ and World Health Organization classification.^10^ The DLBCL cohort and corresponding CEACAM1 immunohistochemistry (IHC) data were previously reported by Xavier et al^3^; that cohort is retained here because the present study extends the prior IHC observations by analyzing CEACAM1 expression in relation to cell-of-origin classification, recurrent somatic mutations, and overall survival in the sequenced subset. The current study additionally evaluates SLL, MZL, and an MM cohort with cytogenetic correlations.

### Immunohistochemistry analysis

Tissue microarrays were created using 1-mm cores from representative tumor areas. Formalin-fixed, paraffin-embedded tissue microarrays were cut at 3 to 4 microns. Routine IHC stains specific for the lymphoma subtype (eg, CD20, BCL2, BCL6, MUM1, CD30) and the anti-CEACAM1 rabbit monoclonal antibody (clone EPR4049; Abcam) were performed using the DISCOVERY Ultra Automated IHC stainer (Ventana Medical Systems). The IHC-stained slides were reviewed by 2 hematopathologists (VRS and JYS). Cases were classified as CEACAM1 positive if more than 50% of tumor cells showed membranous staining above background. Staining intensity was recorded as strong (comparable to the external positive control, colonic epithelium) or weak (detectable membranous staining below the control), and total positivity in Table 1 was defined as the sum of strong and weak categories. Because the primary objective was to compare prevalence across entities, a prespecified dichotomous cutoff was used rather than an H-score. Nonneoplastic lymphoid cells and endothelial elements can show physiologic CEACAM1 staining; scoring was restricted to neoplastic cells to minimize confounding. Cases were independently reviewed by both hematopathologists, and discrepant cases were resolved by joint review to reach a consensus final score.

**Table 1:** CEACAM1 Expression by Immunohistochemistry in Mantle Cell Lymphoma, Other B-Cell Lymphomas, Multiple Myeloma, and Plasmacytoma

Disease	Total No. of cases	Positive expression, No.	Weak expression, No.	CEACAM1-positive cases, %
Mantle cell lymphoma	30	23	4	90
Diffuse large B-cell lymphoma^a^	82	10	9	23
GCB	50	8	7	30
ABC	15	0	2	13
Indeterminate	17	2	0	12
Marginal zone lymphoma	22	3	5	36
Nodal MZL	12	1	2	25
Extranodal MZL	8	2	2	50
Transformed MZL	1	0	1	100
Cutaneous MZL	1	0	0	0
Small lymphocytic lymphoma	30	6	14	67
Multiple myeloma	35	22	1	66
CCND1::IGH positive	20	15	0	75
CCND1::IGH negative	15	7	1	53
Plasmacytoma	2	2	0	100

Abbreviations: ABC, activated B-cell subtype; GCB, germinal center B-cell subtype; MZL, marginal zone lymphoma.

a Diffuse large B-cell lymphoma CEACAM1 immunohistochemistry frequencies previously reported in Xavier et al,^3^ retained here as the reference for the cell-of-origin and mutation correlation analyses.

### Targeted sequencing

Mutational sequencing was reviewed on previously reported cases of DLBCL,^11^ which had corresponding CEACAM1 IHC. Cell-of-origin (COO) was determined by Lymph2Cx and/or Hans algorithm by IHC staining.

### Statistical analysis

Descriptive statistics were employed to summarize the distribution of CEACAM1-positive cases across different lymphoma subtypes and subgroups. The χ^2^ test or Fisher exact test (as appropriate for small cell counts) was used to compare categorical variables, including the proportions of CEACAM1-positive cases between disease subtypes and subgroups. Odds ratios (ORs) are reported with 95% confidence intervals. Statistical significance was set at *P* < .05. Kaplan-Meier survival analysis was used to evaluate the potential association of CEACAM1 expression with overall survival and progression-free survival. All statistical analyses were performed using SPSS (version 26; IBM) and GraphPad Prism (version 9; GraphPad Software).

## RESULTS

Most MCL cases (27/30, 90%) were positive for CEACAM1. Three blastoid/pleomorphic MCL cases were all positive for CEACAM1. The 3 CECAM1-negative cases were conventional-type MCL. Among the other lymphoma entities evaluated, CEACAM1 was positive in 19 of 82 DLBCLs (23%), 8 of 22 MZLs (36%; including 3/12 nodal MZLs, 4/8 extranodal MZLs, 1/1 transformed MZLs, and 0/1 cutaneous MZLs), and 20 of 30 SLLs (67%) (Table 1). The proportion of CEACAM1-positive cases was higher in MCL than in the other lymphoma subtypes combined (90% vs 35%, *P* < .0001).

In DLBCL, COO classification identified 50 germinal center B-cell (GCB), 15 activated B-cell (ABC), and 17 indeterminate cases, and CEACAM1 positivity was more frequent in GCB than ABC DLBCL (30% vs 13%, *P* = .023).

**Figure 1:**
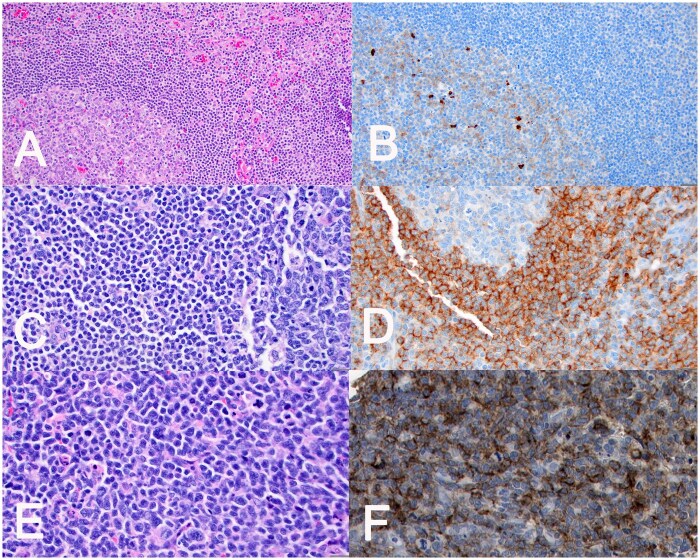
Morphology and CEACAM1 immunohistochemistry (IHC) in reactive tonsil (A, B), conventional mantle cell lymphoma (MCL) (C, D), and blastoid/pleomorphic mantle cell lymphoma (E, F).

**Figure 2:**
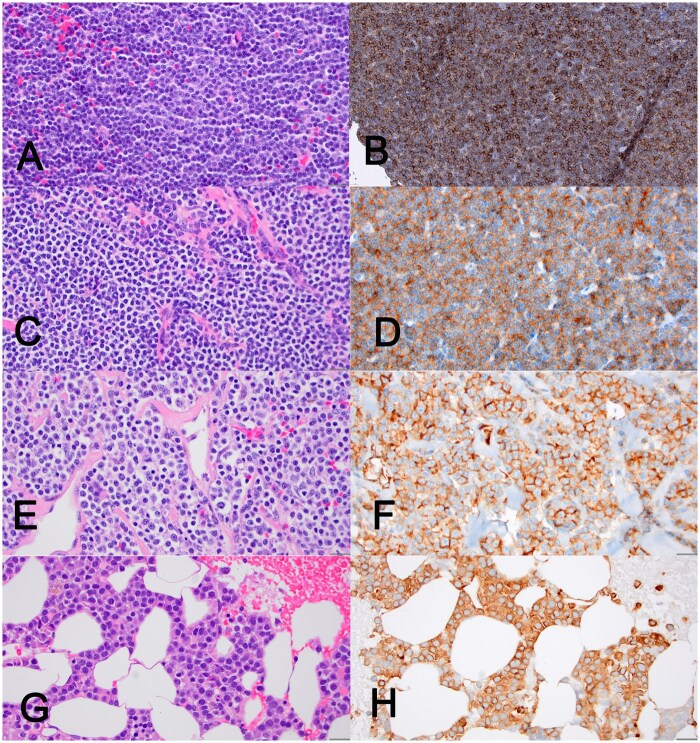
Morphology and CEACAM1 immunohistochemistry (IHC) in small lymphocytic lymphoma (SLL), nodal marginal zone lymphoma (NMZL), transformed marginal zone lymphoma, and multiple myeloma. (A) Hematoxylin and eosin (H&E) and (B) CEACAM1 immunohistochemistry (IHC) of SLL. (C) H&E and (D) CEACAM1 IHC of nodal MZL. (E) H&E and (F) CEACAM1 IHC of MZL with increased large cells. (G) H&E and (H) CEACAM1 IHC of multiple myeloma.

We evaluated the expanded MM cohort to assess associations between CEACAM1 expression and conventional karyotype and fluorescence in situ hybridization findings. CEACAM1 was expressed in 23 of 35 MM cases (66%; 22 strong, 1 weak) and in 2 of 2 plasmacytomas. CEACAM1 positivity did not significantly differ by t(11;14)/CCND1::IGH status, although a nonsignificant trend toward higher expression was observed in t(11;14) cases (15/20, 75%) compared with non-t(11;14) cases (8/15, 53%; OR, 2.6; Fisher exact *P* = .28). No significant associations were identified between CEACAM1 expression and del17p/TP53 loss (33% vs 76%; OR, 0.15; *P* = .13); gain of 1q/CKS1B (56% vs 71%; *P* = .66); hyperdiploidy with trisomies of chromosomes 5, 9, and 15 (57% vs 69%; *P* = .66); monosomy 13 or del(13q) (55% vs 75%; *P* = .40); MYC rearrangement; t(4;14)/FGFR3::IGH; t(14;16)/IGH::MAF; or karyotypic complexity (Figure S1). These findings should be interpreted with caution, given the small sample size. Paired hematoxylin and eosin and CEACAM1 IHC images are shown in Figure 1 for reactive tonsil (panels A–B), conventional/classic MCL (panels C–D), and blastoid/pleomorphic MCL (panels E–F), as well as in Figure 2 for SLL (panels A–B), nodal MZL (panels C–D), transformed MZL (panels E–F), and MM involving bone marrow (panels G–H). In reactive tonsil (Figure 1A, B), CEACAM1 IHC showed weak staining of GCBs, with negative or below-detection staining of mantle zone B cells and interfollicular T cells; plasma cells, in contrast, showed strong membranous CEACAM1 expression. In MCL, both conventional/classic (Figure 1C, D) and blastoid/pleomorphic (Figure 1E, F) variants showed strong, diffuse membranous CEACAM1 expression. In bone marrow involved by MM (Figure 1G, H), neoplastic plasma cells showed strong membranous CEACAM1 staining. In Figure 2, SLL showed membranous CEACAM1 expression (panels A–B), nodal MZL also showed membranous expression (panels C–D), and transformed MZL showed membranous CEACAM1 staining (panels E–F).

### COO and mutation analysis in DLBCL

**Figure 3:**
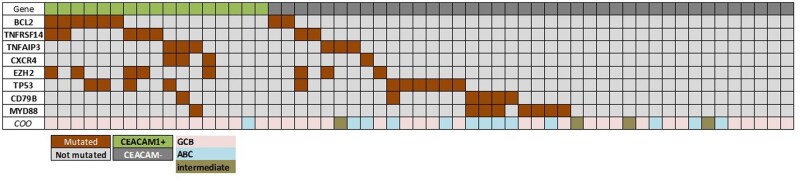
Mutational landscape of de novo diffuse large B-cell lymphoma. CEACAM1-positive cases were frequent in germinal center B-cell diffuse large B-cell lymphoma, showing enriched mutations in *BCL2* and *TNFRSF14* compared to CEACAM1-negative cases. Additional recurrent genes displayed include *TNFAIP3*, *CXCR4*, *EZH2*, *TP53*, *CD79B*, and *MYD88*.

Targeted mutational data were available for 57 of the 82 DLBCL cases. Within this sequenced subset, there were 40 GCB, 13 ABC, and 4 intermediate/unclassified cases, determined by Lymph2Cx and/or the Hans algorithm. Within this sequenced DLBCL subset, CEACAM1 expression was not significantly associated with overall survival by Kaplan-Meier analysis (log-rank *P* > .05) (Figure S2). Kaplan-Meier analyses were not performed for the other lymphoma subtypes because of limited follow-up data and small subgroup sizes. CEACAM1 expression was significantly associated with mutations in *BCL2* (OR, 9.3; 95% CI, 1.6-52.1; *P* = .009) and *TNFRSF14* (OR, 7.1; 95% CI, 1.3-43.3; *P* = .03) (Figure 3). Other recurrent mutations evaluated are shown in Figure 3, including *TNFAIP3*, *CXCR4*, *EZH2*, *TP53*, *CD79B*, and *MYD88*.

## DISCUSSION

Mantle cell lymphoma remains a clinically aggressive lymphoma with frequent relapses, underscoring the need for actionable surface targets and biomarkers.^1‐3^^,^^6^^,^^12^ CEACAM1 has emerged as a modulator of oncogenic B-cell receptor signaling in MCL and may also contribute to immune regulation in the tumor microenvironment.^3^^,^^13‐16^ In this study, we provide a comparative survey of CEACAM1 membranous expression across multiple B-cell lymphoma subtypes and MM to inform prioritization of CEACAM1-directed strategies. The present study includes classic Hodgkin lymphoma (CHL), DLBCL, and follicular lymphoma (FL) cohorts previously reported in Xavier et al,^3^ which are incorporated here for cross-entity comparison, together with newly analyzed disease groups.

In a mechanistic study by Xavier et al^3^ using murine models and super-resolution confocal microscopy, CEACAM1 was found to promote reorganization of the actin cytoskeletal network forming a central scaffold for lipid rafts, which enabled oncogenic BCR signaling in MCL. Many studies have shown similar interactions between the formation of lipid rafts with B-cell signaling and promotion of B-cell activation, but none have shown the involvement of CEACAM1 in this process.^17‐19^ In contrast, Lobo et al^20^ found that CEACAM1 may act as an inhibitory B-cell receptor.^19^ Another study by Tsugawa et al^13^ also found that CEACAM1 activation resulted in suppression of BCR signaling.^20^ Despite some controversy, there is agreement that CEACAM1 has a number of different mechanisms depending on the functional adaptor, which is bound by CEACAM1, and the range of substrates with which the functional adaptor can interact.^21^ Additionally, the intracytoplasmic part of the CEACAM1 membrane receptor is said to impart either inhibitory or noninhibitory signals depending on the size of the cytoplasmic tail.^22^

Our study demonstrates frequent CEACAM1 expression in MCL (90%) and in subsets of other B-cell malignancies, including SLL (67%), MM (66%), and MZL (36%). Within MZL, CEACAM1 expression was identified across nodal, extranodal, and transformed subtypes, with the highest frequencies in the extranodal subtype, although the numbers in some subgroups remain small. In MM, CEACAM1 expression was present in 66% of cases. Across the expanded MM cohort with available conventional karyotype and fluorescence in situ hybridization data, we did not identify a statistically significant association between CEACAM1 expression and any specific cytogenetic abnormality, but a nonsignificant trend toward higher CEACAM1 expression in t(11;14) cases (75% vs 53%, *P* = .28) and toward lower expression in cases with *TP53* loss (33% vs 76%, *P* = .13) was observed but should be interpreted as hypothesis-generating given the limited sample size.

Among DLBCLs, CEACAM1 expression was more frequent in the GCB subtype than the ABC subtype (30% vs 13%). Within the sequenced subset, CEACAM1 positivity was associated with *BCL2* and *TNFRSF14* mutations, while additional recurrent mutations (*TNFAIP3*, *CXCR4*, *EZH2*, *TP53*, *CD79B*, *MYD88*) are summarized in Figure 3. Because the DLBCL cohort was enriched for GCB cases, these findings should be interpreted cautiously, particularly with respect to subtype comparisons and survival analyses.

The associations between CEACAM1 expression and *BCL2* and *TNFRSF14* mutations in our DLBCL cohort warrant further mechanistic consideration. *BCL2* mutations and the t(14;18) translocation are characteristic of GCB DLBCL and follicular lymphoma, where BCL2 overexpression supports antiapoptotic survival in cells maintaining or arising from a germinal center program.^23^ CEACAM1 can upregulate BCL2 expression via PI3K/AKT in monocytes and can promote B-cell survival through BTK/SYK/NFkB, and it is conceivable that in the context of neoplastic B-cells, CEACAM1 adopts an activating rather than an inhibitory signaling mode, as seen in MCL.^3^^,^^24^  *TNFRSF14* (HVEM) is recurrently mutated in GCB DLBCL and follicular lymphoma, and loss-of-function mutations are thought to disrupt the HVEM-BTLA inhibitory axis, attenuate T-cell suppressive signals, and shift the tumor microenvironment toward immune evasion.^25^^,^^26^ CEACAM1, through its ITIM-containing long isoform, also delivers inhibitory signals in immune cells and contributes to T-cell exhaustion when coexpressed with TIM-3, suggesting a parallel role in immune-evasive checkpoint biology.^25^ The co-occurrence of CEACAM1 expression with *BCL2* and *TNFRSF14* mutations is consistent with a model in which CEACAM1 contributes to both intrinsic survival and microenvironmental immune escape in GCB-derived B-cell neoplasms. These observations are speculative and hypothesis-generating, requiring validation in larger sequenced cohorts.

Interpretation of CEACAM1 expression in relation to the COO of individual B-cell lymphomas should be cautious because several entities, including MCL, SLL, and MZL, have biologically heterogeneous origins reported in the literature. In addition, CEACAM1 expression at the protein level in our reactive tonsil showed weak staining in GCBs and negative or below-detection staining in mantle zone and interfollicular T cells, with strong staining limited to plasma cells. This pattern is concordant with the high frequency of CEACAM1 positivity in our MM cohort but is discordant with the high CEACAM1 expression observed in MCL, the mantle zone counterpart. One potential interpretation is that mantle zone B cells in resting tonsil express CEACAM1 below the sensitivity threshold of IHC on formalin-fixed, paraffin-embedded tissue, whereas chronic B-cell receptor signaling and oncogenic dysregulation in MCL drive CEACAM1 to readily detectable levels. A limitation of the present study is that no flow cytometric assessment of CEACAM1 expression in peripheral blood or bone marrow was performed. In the present study, we did not have immunoglobulin heavy chain variable (IGHV) mutation status or transcriptomic correlates, and we did not separately score SLL proliferation centers, which limits mechanistic inference regarding lineage and BCR-signaling state. Future studies that integrate IGHV mutation status, messenger RNA expression, and clinical correlates, including the CEACAM1-negative subset of MCL, will be important to clarify biologic and therapeutic implications.

CEACAM1 may influence lymphoma biology through at least 2 mechanisms, such as modulation of BCR signaling in tumor cells and checkpoint-like functions that contribute to T-cell dysfunction in the tumor microenvironment.^3^^,^^13‐15^ Therapeutic antibodies targeting CEACAM1 are in clinical investigation in solid tumors, supporting further exploration of whether CEACAM1-directed strategies may have relevance in CEACAM1-expressing lymphoid neoplasms. In summary, this study demonstrates frequent CEACAM1 expression across several B-cell malignancies, particularly MCL, SLL, MM, and a subset of MZL cases, supporting further work to define clinical utility and therapeutic targeting.

## Supplementary Material

aqag078_Supplementary_Data

## Data Availability

The data underlying this article cannot be shared publicly due to patient privacy and institutional data-use restrictions. De-identified data may be made available on reasonable request to the corresponding author, subject to institutional approval and applicable ethical and regulatory requirements.
