# Supplementary material for: CEACAM1 expression by immunohistochemistry in B-cell lymphomas and plasma cell myeloma
Source: Am J Clin Pathol. 2026 Jun 29;165(6):aqag078. doi: 10.1093/ajcp/aqag078 (PMC13312280; doi:10.1093/ajcp/aqag078)
Supplement: aqag078_Supplementary_Data [file aqag078_supplementary_data.docx]

Supplemental Material


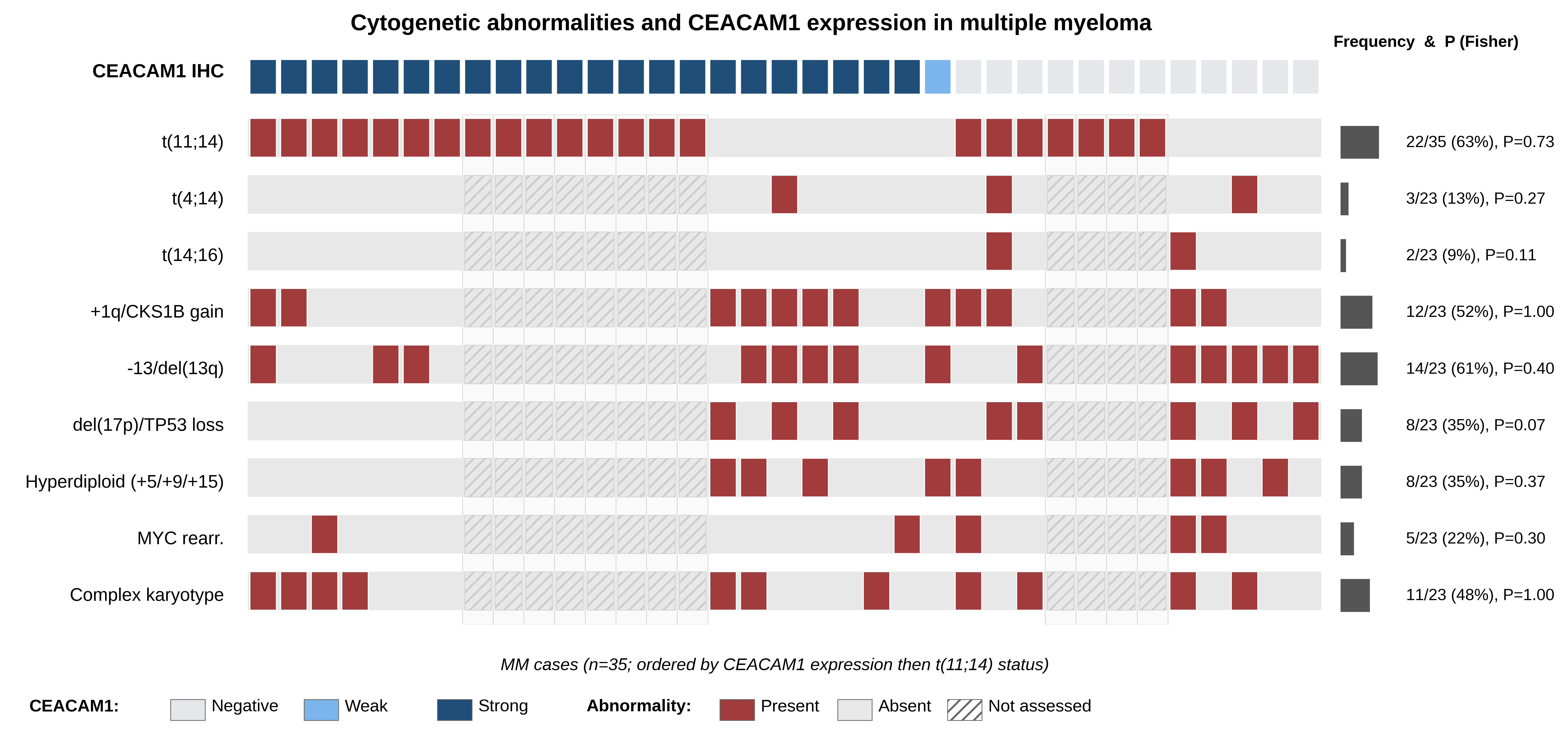


**Supplemental Figure 1.** *Cytogenetic abnormalities and CEACAM1 expression in 35 cases of multiple myeloma.* Each column represents one MM case, ordered by CEACAM1 IHC status (top track: dark blue strong, light blue weak, gray negative) and then by t(11;14) status. Red cells indicate that the abnormality was detected in that case; gray indicates not detected. Hatched cells (rightmost columns) mark cases ascertained for t(11;14)/CCND1::IGH only without complete karyotype/FISH workup, so other abnormalities are reported as 'not assessed' rather than absent.


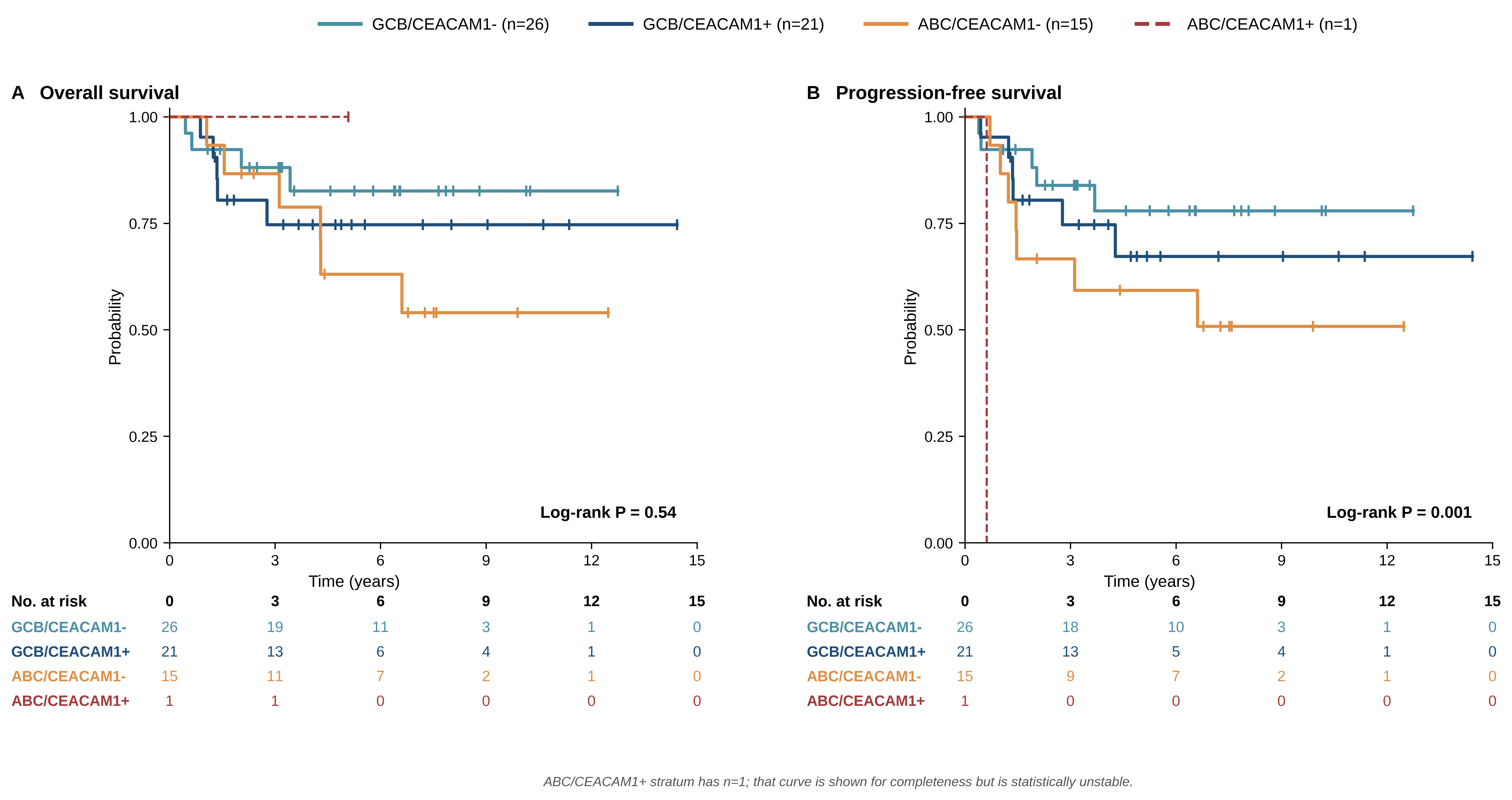


**Supplemental Figure 2.** *Kaplan-Meier estimates of overall and progression-free survival in DLBCL stratified by cell-of-origin (COO) and CEACAM1 IHC status.* Sixty-three patients with COO DLBCL (47 GCB, 16 ABC) and complete survival data are included; intermediate/unclassified cases were excluded. Tick marks denote censoring. P values are global four-stratum log-rank tests. The ABC/CEACAM1+ stratum contains a single patient (shown as a dashed line); a sensitivity analysis dropping that patient gave OS log-rank P = 0.40 and PFS log-rank P = 0.28, suggesting the apparent four-stratum PFS difference is driven by this small group rather than by an independent effect of CEACAM1 within the ABC subset.
